# Supplementary material for: Water−Processed Organic Solar Cell with Efficiency Exceeding 11%
Source: Polymers (Basel). 2022 Oct 9;14(19):4229. doi: 10.3390/polym14194229 (PMC9573733; doi:10.3390/polym14194229)
Supplement: Supplementary file 1 [file polymers-14-04229-s001.zip › polymers-1928722-SI.pdf]

Water−Processed Organic Solar Cell with Efficiency Exceeding 11%

Chen Xie *, Songqiang Liang, Guangye Zhang and Shunpu Li *

College of New Materials and New Energies, Shenzhen Technology University, Shenzhen 518118, China

* Correspondence: xiechen@sztu.edu.cn (C.X.); lishunpu@sztu.edu.cn (S.L.)

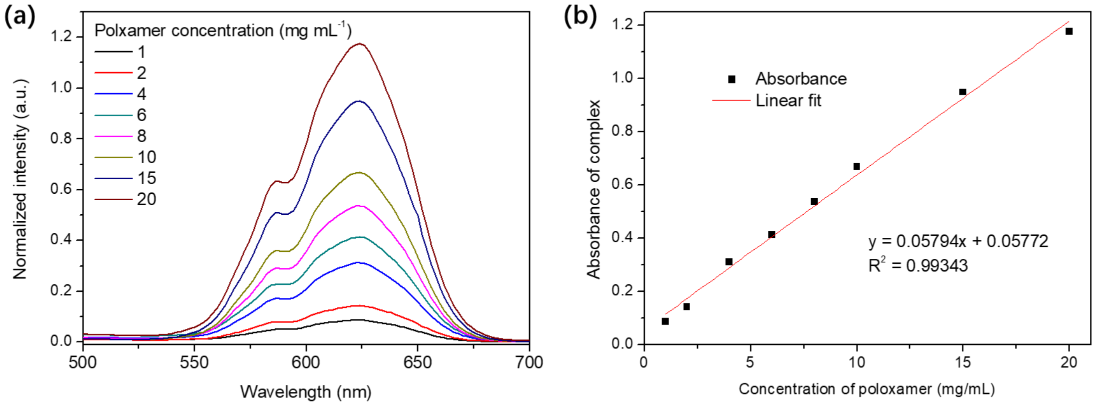

Figure S1. (a) Absorption and (b) calibration curve used to determine concentration of free poloxamer during centrifugal washing. Poloxamer and cobalt thiocyanate formed a dark blue complex (absorbance at 623 nm).

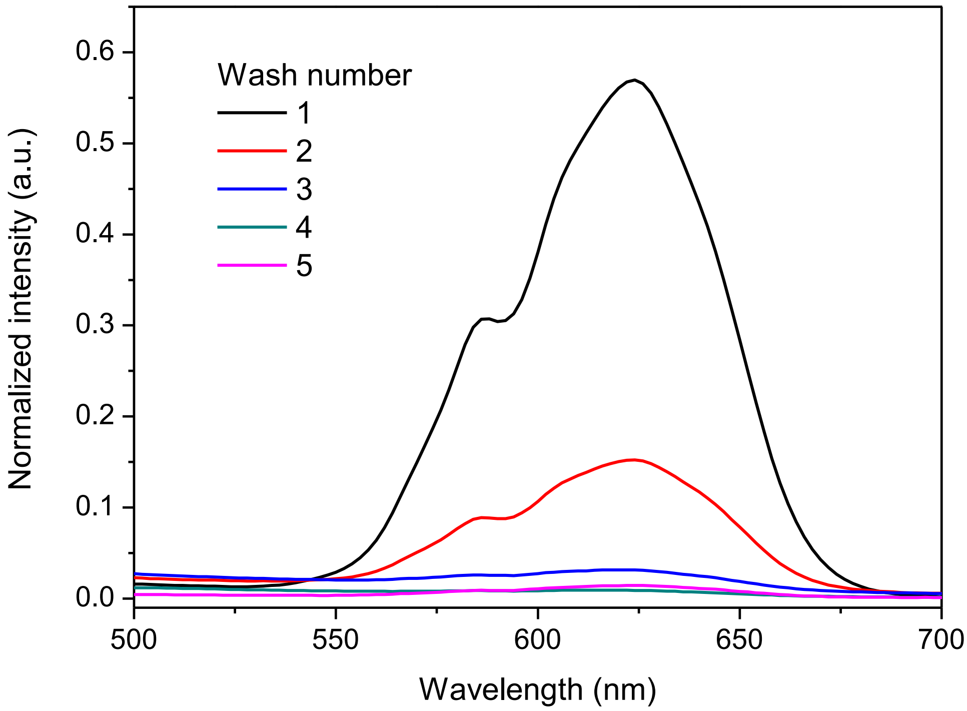

Figure S2. Absorption used to determine the concentration of poloxamer in filtrates collected from each centrifugal washing step.

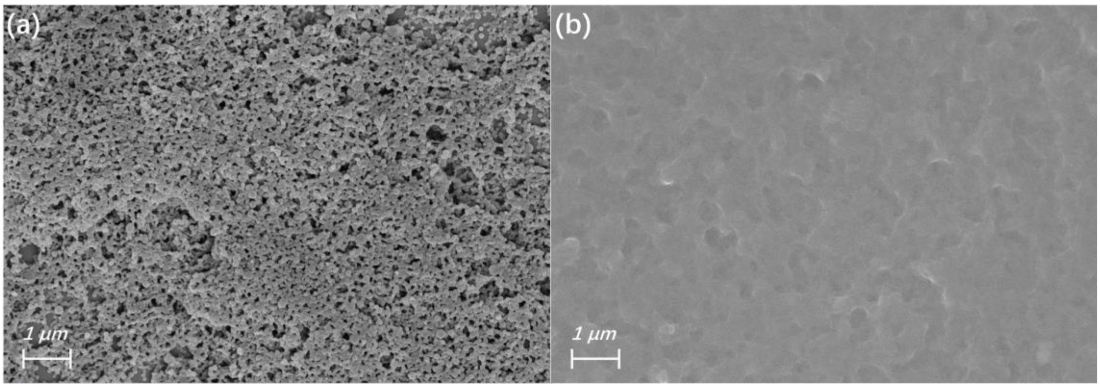

Figure S3. SEM images of PM6:BTP−eC9 NP films: (a) as cast; (b) 100 ºC annealing for 10 minutes. .

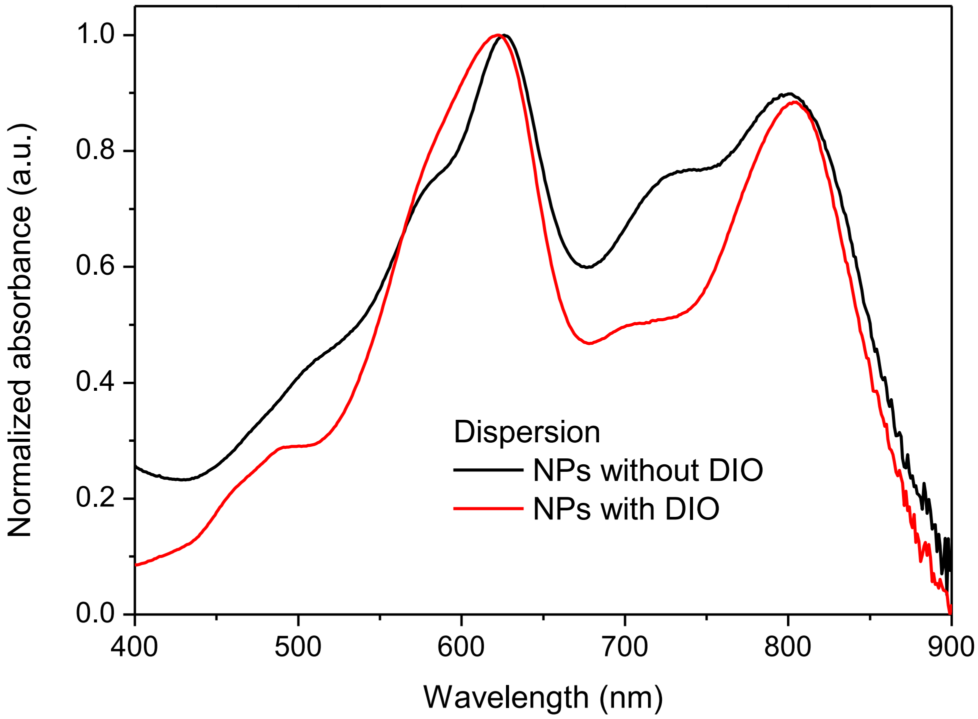

Figure S4. Normalized UV−vis absorption spectra of PM6:BTP−eC9 ssNP in water dispersion.

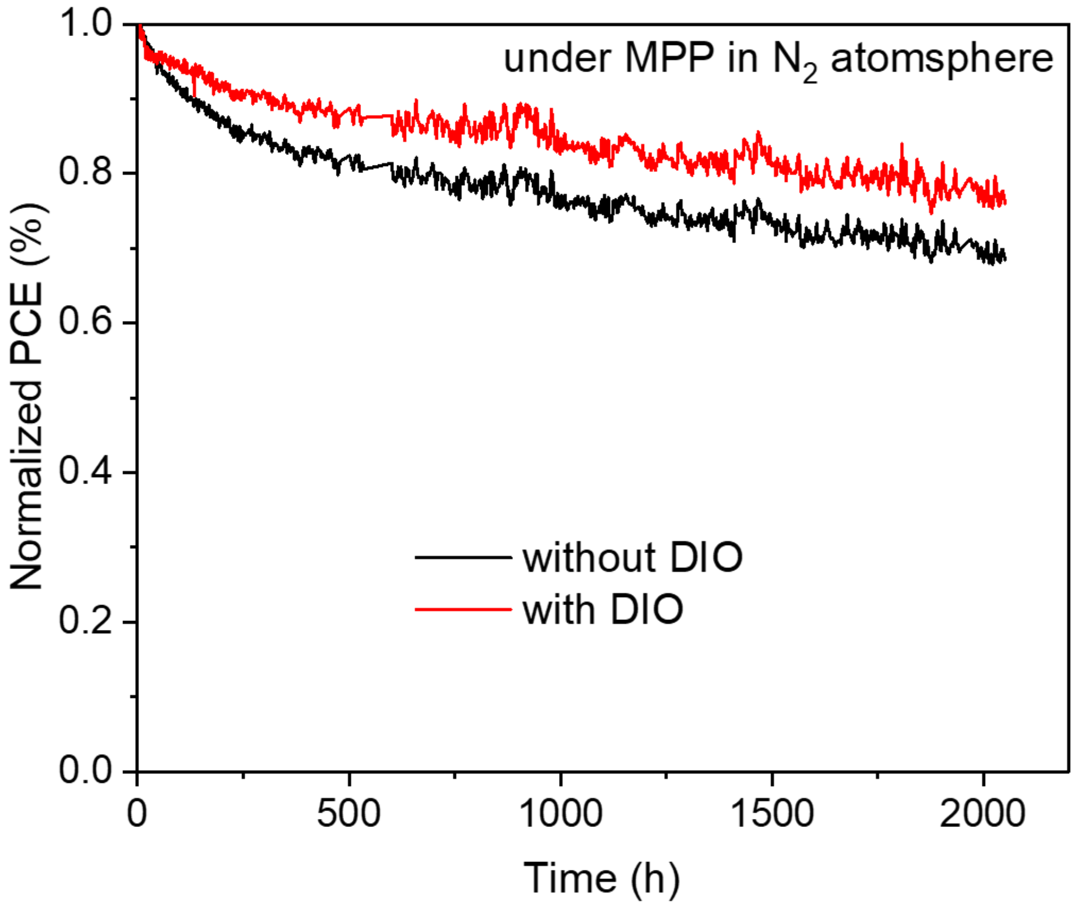

Figure S5. Normalized PCE decay of PM6:BTP−eC9 ssNP processed solar cells under maximum power point (MPP) tracking in N_2 atmosphere.

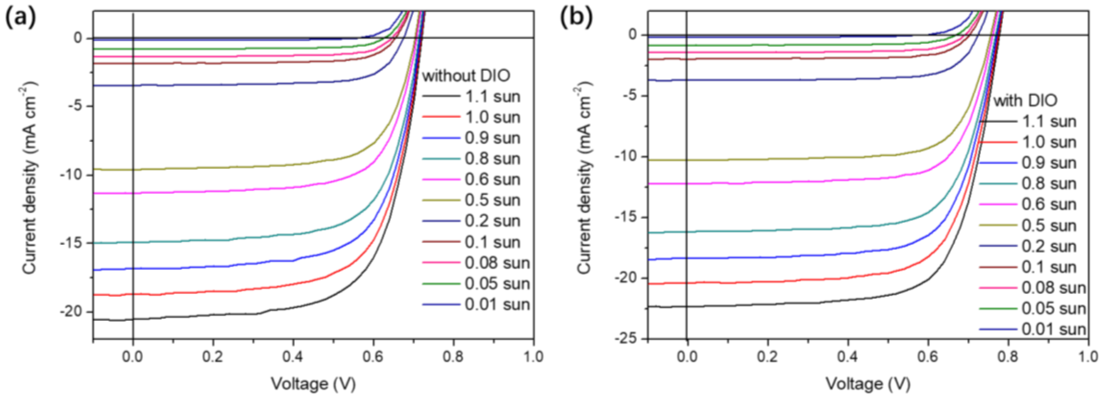

Figure S6. J−V characteristics of PM6:BTP−eC9 ssNP processed solar cells (a) without DIO and (b) with DIO under different light intensities.
